# Investigating causes and risk factors of pre-chemotherapy viremia in acute lymphoblastic leukemia pediatric patients

**DOI:** 10.1007/s15010-022-01878-9

**Published:** 2022-07-25

**Authors:** Nivin Abdel-azim, Lamiaa Fadel Alkilany, Zeinab Korany Hassan, Noha Gaber

**Affiliations:** 1 Cancer Biology Department, South Egypt Cancer Institute, Assiut University, Assiut, 71516 Egypt; 2 Virology and Immunology Unit, Cancer Biology Department, National Cancer Institute, Cairo University, Cairo, Egypt; 3 Clinical Pathology Department, South Egypt Cancer Institute, Assiut University, Assiut, Egypt

**Keywords:** Viremia, Co-detection, ALL, Polyoma BK, Herpes simplex

## Abstract

**Background:**

Leukemia patients are immune-compromised even before starting chemotherapy because the malignant cells invade the bone marrow and destroy WBC precursors. Leukemic patients are more susceptible to infection by a wide range of microorganisms. Viral infections and reactivations are common and may result in severe complications. The aim of this study is to investigate different causes of viremia in ALL pediatric patients as well as the clinical and the laboratory characteristics associated with viral infections.

**Methods:**

Qualitative real-time PCR was used to detect (polyoma BK, parvo B19 and herpes simplex virus) DNA in the blood of ALL patients and routine hospital records were used to provide the data of hepatitis B & C virus infection.

**Results:**

Polyoma BK was the most common detected virus (51.2%) followed by herpes simplex (30.2%)**.** Viremia by single virus was found in 16 (37.2%) cases, while viremia by multiple viruses was found in 15 (34.8%) cases**.** The most frequent co-detected viruses were herpes simplex and polyoma BK (11.6%) followed by herpes simplex, parvo B19 and polyoma BK (9.3%)**.**

**Conclusion:**

There is a high frequency of viremia by single virus and viremia by multiple viruses at the time of diagnosis of acute lymphoblastic leukemia in pediatric patients admitted to South Egypt Cancer Institute (SECI) compared to studies in other regions. Polyoma BK is the most common detected virus and is mainly associated with lymphopenia. It was also significantly associated with herpes simplex viremia. HCV infection was associated with increased incidence of CNS leukemia.

**Supplementary Information:**

The online version contains supplementary material available at 10.1007/s15010-022-01878-9.

## Introduction

Acute lymphoblastic leukemia (ALL) is documented now to be the most common childhood malignancy, accounting for 25% of all pediatric cancers [1]. Leukemia patients are immunocompromised either due to the disease state which involves clonal expansion of abnormal lymphoid progenitors that are undifferentiated and abnormally functioning. They invade the bone marrow, peripheral blood, and extramedullary sites, [2] or immunocompromised due to chemotherapy induced immune suppression. Immunocompromised patients are at high risk of viral reactivation or new virus infections [3].

Latent virus reactivation either solely or simultaneously can produce serious consequences [4], so prompt and precise diagnosis is important for starting treatment at the appropriate time and preventing progression to overt disease.

The common latent viruses that can be reactivated in immunocompromised state are human herpes family and polyoma family viruses [5, 6]. Parvo B19 causes persistent infections in immunocompromised patients [7]. Chronic liver infections (Hepatitis B and hepatitis C viruses) are common in leukemia patient [8, 9]. Egypt used to be the country with the highest prevalence of HCV infection in the world.

With the introduction of effective direct-acting antiviral agents (DAAS) in 2014 to treat HCV infection, a national strategy was set by the National Committee for Control of Viral Hepatitis (NCCVH) and launched massive screening programs to detect unidentified cases and provide unpaid treatment for all cases [10].

Frequency of viral infections and their risk factors in leukemia are less investigated among Egyptian children. The aim of this study is to investigate causes of viremia in ALL pediatric patients as well as the clinical and the laboratory characteristics associated with viral infections.

## Materials and methods

The study included 43 pediatric acute lymphoblastic leukemia patients admitted to Pediatric Oncology Department at our institute. The study included ALL pediatric patients under the age of 18 either first presentation cases or relapsed cases. None of them was presented more than once. Bone marrow transplant patients and patients who were under chemotherapy were excluded from the study. The patients were evaluated for the presence of leucopenia (leucocytic count < 5 × 10^3^/μl, lymphopenia (lymphocytic count < 3 × 10^3^/μl), moderate and severe anemia < 10 g/dl, thrompocytopenia (platelet count < 150 × 10^3^/μl), abnormal creatinine (normal values 0.5 to 1.0 mg/dl for children aged 3 to 18 years and 0.3 to 0.7 mg/dl for children younger than age 3), abnormal liver enzymes (Normal liver enzymes SGOT [serum glutamic oxaloacetic transaminase] up to 31 IU/l and SGPT [serum glutamic pyruvic transaminase] up to 45 IU/l). Routine hospital serological viral tests were done for hepatitis C by HCV antibody (Monolisa HCVAg-Ab Ultra V2, BIO-RAD, USA) and hepatitis B by detecting hepatitis B surface antigen (HBsAg) test (Monolisa HBs Ag Ultra, BIO-RAD, USA) as well as quantitative PCRtesting. Informed consent was obtained from the parents of the children or their legal guardians according the Declaration of Helsinki (The Code of Ethics of the World Medical Association) for experiments involving humans and the study was approved by Institutional Review Board of South Egypt Cancer Institute (SECI-IRB, approval number 496/IORG0006563).

### Sampling

One ml of blood is withdrawn in EDTA tube then centrifuged for plasma separation; the plasma was stored in -70.

### Viral DNA extraction

The stored plasma samples were thawed and viral DNA was extracted by Gene JET Viral DNA and RNA Purification Kit (Thermofisher Scientific).

### First step of polyoma virus detection

Polyoma virus detection was done by semi-nested PCR. For the amplification of target genes, PCR was run in two separate steps. First step conventional PCR amplification was performed with 2 μl of extracted DNA, 10 μl DreamTaq Green PCR master mix (Thermo fisher Scientific), 1 μl forward outer primer, and 1 μl reverse outer primer in a final volume of 20 μl in ARKTIK thermal cycler (Thermofisher Scientific).

Thermal cycler conditions were adjusted as follows: a first denaturing cycle at 94 °C for 5 min, followed by 40 cycles of amplification defined by denaturation at 94 °C for 30 s, 55 °C annealing temperature for 45 s, and extension at72 °C for 1 min. A final extension cycle of 72 °C for 5 min was included. The reaction was visualized on 2% agarose gel stained with ethidium bromide and visualized under ultraviolet light. In a second-round PCR, 1 μl of the 1:10-diluted first round PCR product was amplified with the common forward outer primer and specific polyoma BK primer under the same real-time PCR volumes and conditions of herpes simplex virus and parvo B19 virus.

### Detection of herpes simplex virus and parvo B19 virus and second step of semi-nested PCR of polyoma virus

A total reaction volume of 20 µl was used for the real-time qPCR. It included 10 µl of Maxima SYBER Green qPCR master mix (Thermofisher Scientific), 0.5 µl of forward primer (10 pmol/ml), 0.5 µl of reverse primer (10 pmol/ml). Primers are listed in Table 1 and 5 µl of extracted DNA as a template for parvo B19 and herpes virus detection and1 μl of the 1:10-diluted product of the first round PCR as a template for polyoma BK detection. The reaction mixture was used in a qualitative real-time PCR run. The cycling conditions for the three reactions were as follows: initial denaturation cycle of 95 °C for 10 min; 45cycles of 95 °C for 15 s, 58 °C for 30 s, 72°for 30 s; and final extension of 60 °C for 15 s, with a gradual increase to 95 °C in 30 min. Applied Biosystems 7500 real-time system was used for amplification.

**Table 1:** Primers used for real-time PCR

Virus	Primer sequences	Accession number
Parvo 19 V-F Parvo B19V-R	5ʹ-ACCAGTTCAGGAGAATCAT-3ʹ 5ʹ-CCCACACATAATCAACCC-3ʹ	
HSV1/2-F HSV1/2-R	5ʹ-CCGGAGAGGGACATCCAGGACTT-3ʹ 5ʹ-GGGCCATGAGCTTGTAATACACCGT-3ʹ	
Polyoma outer–F Polyoma outer-R	5-AAGTCT TTAGGG TCTCTAC-3 5 -GTG CCA ACCTATGGA ACAGA-3	
Polyoma specific BK-R	5′-GAGTCCTGGTGGAGTTCC-3	

*HSV* herpes simplex virus, *F* forward primer, *R* reverse primer

Positive controls from previously confirmed positive patients were included in each run

### Statistical analysis

The analysis of the data was carried out using the IBM SPSS 20.0 statistical package software (IBM; Armonk, New York, USA). The Venn diagram was constructed by using Microsoft Excel. Normality of the data was tested using the Shapiro–Wilk test. Binary logistic regression analysis was used to see the combined effect of different independent variables on the target (dependent variable). *P* value less than 0.05 was considered significant.

## Results

This study included 43 ALL patients with median age 7 years. It included 26 (60.5%) males and 17 (39.5%) females. Thirty three percent (33%) of the ALL cases were of B-cell lineage. Seventy six percent (76.7%) of the patients had relapsed disease but only 23.3% of patients were first presentation cases. The clinical and laboratory characteristics of the patients are illustrated in Table 2.

**Table 2:** Demographical, clinical and laboratory characteristics of ALL cases

	All (*N* = 43)
	*N* (%)/ Median (Range)
Age (y)	7 (2–15)
Sex	
Male	26 (60.5%)
Female	17 (39.5%)
Lineage of ALL	
B-cell	33 (76.7%)
T-cell	10 (23.3%)
Status of presentation	
Denovo	10 (23.3%)
Relapse	33 (76.7%)
CNS involvement	
Present	15 (34.9%)
Absent	28 (65.1%)
Urinary symptoms (dysuria or haematuria)	4 (9.3%)
GIT mucositis	11 (25.6%)
Hb (g/dl)	11 (5.5–14)
Platelets (× 10^3^/mL)	222 (24–905)
WBC count (× 10^3^/mL)	6.3 (3–390)
Leukopenia	18 (41.9%)
Lymphocytic count (× 10^3^/mL)	3 (0.3–286)
Lymphopenia	13 (30.2%)
Creatinine (mg/dl)	0.3 (0.1–1.2)
High Creatinine	2 (4.7%)
SGOT liver enzyme (AST)	38 (15–243)
High SGOT	30 (69.8%)
SGPT liver enzyme (ALT)	34 (7–230)
High SGPT	15 (34.9%)

*AST* Aspartate aminotransferase, *ALT* Alanine aminotransferase, *CNS* central nervous system, *GIT* gastrointestinal tract, *Hb* hemoglobin, *SGPT* serum glutamic pyruvic transaminase, *SGOT* Serum glutamic oxaloacetic transaminase, *WBC* white blood cell

Of the 43 patients, 31 (72.1%) cases were tested positive for at least one of the viruses analyzed. Single virus detection was found in 16 (37.2%) cases, while viremia by multiple viruses was found in 15 (34.8%) cases. Polyoma BK was the most common detected virus (51.2%) followed by herpes simplex (30.2%).

Only one case was positive for five different viruses. The frequency of detection of each virus is presented in Table 3.

**Table 3:** Frequency of viral infections/reactivations among ALL cases

	All (*N* = 43)	
	*N*	%
Polyoma BK	22	51.2
Herpes simplex I&II	13	30.2
Human parvo B19	9	20.9
HCV	9	20.9
HBV	2	4.7
Viremia by multiple viruses		
No viral detection	12	27.9
1 virus detected	16	37.2
2 viruses detected	8	18.6
3 viruses detected	6	14.0
5 viruses detected	1	2.3

*HCV* hepatitis C virus, *HBV* hepatitis B virus

Due to co-infections, the percentages equal more than 100

The most frequent co-detected viruses were herpes simplex and polyoma BK followed by herpes simplex, parvo B19 and polyoma BK co-virus detection (9.3%) (Table 4).

**Table 4:** Frequency of viremia by single virus and viremia by multiple viruses in the blood of ALL cases

	All (*N* = 43)	
	*N*	%
Viremia by multiple viruses		
Herpes simplex, polyoma BK	5	11.6
Herpes simplex, parvo B19, polyoma BK	4	9.3
Parvo B19, polyoma BK	2	4.7
HCV, herpes simplex, polyoma	2	4.7
HCV, HBV, herpes simplex, parvo B19, Polyoma BK	1	2.3
Herpes simplex, parvo B19	1	2.3
Viremia by single virus		
Polyoma BK	8	18.6
HCV	6	14.0
HBV	1	2.3
Parvo B19	1	2.3

*HCV* hepatitis C virus, *HBV* hepatitis B virus

**Fig. 1:**
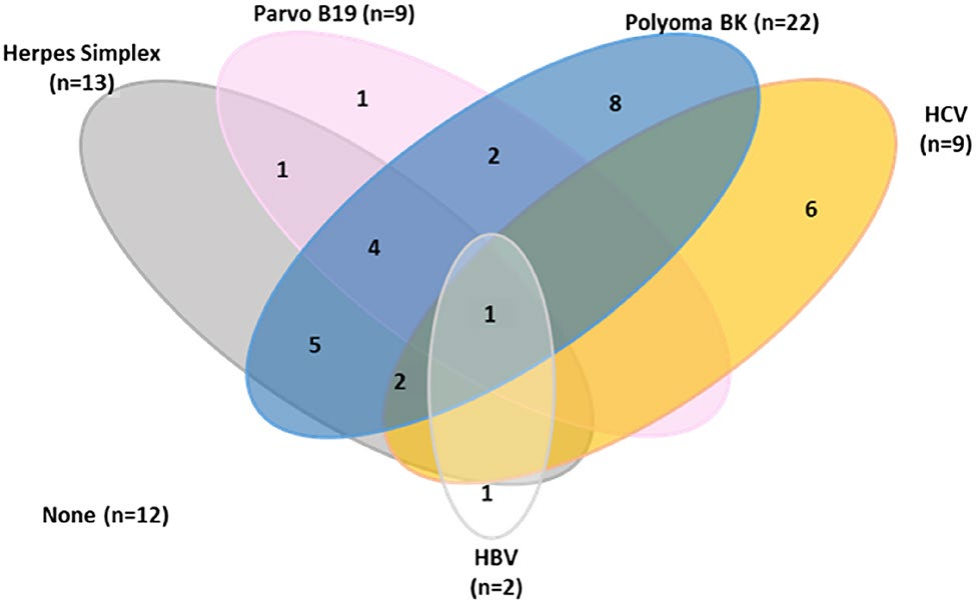
Single viral detection and co-virus detections in the blood of ALL patients. Venn diagram summarizes the distribution of viruses in samples. Viral co-detections are seen overlapping while single viral detection are represented at the end of the diagram (*N* = 16). There were 12 samples with no virus detection

Because viral co-detections were overlapping, we designed Venn diagram for easy interpretation (Fig. 1).

The univariate analysis showed that polyoma BK virus viremia was associated with parvovirus B19 (0.013) and herpes simplex virus detection was associated with polyoma BK detection (*p* value 0.004). The multivariate analysis confirmed the significant association between polyoma BK viremia and herpes simplex viremia (*p* value 0.004).

The univariate analysis showed that polyoma BK virus infection or reactivation was significantly associated with occurrence of lymphopenia (*p* value 0.033) but was not associated with impaired renal function. Parvo B19 virus infection was not associated with cytopenia. HCV infection was associated with a five-fold increased odds of having CNS leukemia than those without hepatitis C infection [OR = 5.56 (95% confidence (1.14–27.01) (*p* value 0.034) as illustrated in Table 5.

**Table 5:** Binary logistic regression analysis of factors associated with viral infections/reactivations in ALL cases

	Herpes simplex		Parvo B19		Polyoma BK		HCV		HBV	
Variables (Univariate analysis)	OR (95% CI)	*p* value	OR (95% CI)	*p* value	OR (95% CI)	*p* value	OR (95% CI)	*p* value	OR (95% CI)	*p* value
Clinical data										
Age (y)	1.13 (0.94–1.36)	0.20	1.22 (0.99–1.52)	0.07	1.04 (0.88–1.23)	0.67	1.09 (0.89–1.34)	0.39	1.66 (0.88–3.12)	0.12
Sex (male)	1.07 (0.28–4.05)	0.93	2.76 (0.5–15.29)	0.24	0.4 (0.11–1.41)	0.16	1.4 (0.3–6.56)	0.67	NE	
B-cell	0.56 (0.13–2.47)	0.45	0.52 (0.1–2.61)	0.43	0.36 (0.08–1.63)	0.18	0.52 (0.1–2.61)	0.43	0.28 (0.02–4.95)	0.39
Denovo cases	0.99 (0.21–4.61)	0.99	1.93 (0.38–9.71)	0.43	0.94 (0.23–3.88)	0.93	0.35 (0.04–3.18)	0.35	NE	
CNS involvement	2 (0.52–7.65)	0.31	0.92 (0.19–4.34)	0.91	0.32 (0.09–1.2)	0.09	5.56 (1.14–27.01)	0.034*	1.93 (0.11–33.21)	0.65
Other viral infections(or reactivations)										
Herpes simplex			7.71 (1.53–38.83)	0.013*	24 (2.72–211.6)	0.004*	1.2 (0.25–5.77)	0.82	2.42 (0.14–41.87)	0.54
Parvo B19	7.71 (1.53–38.83)	0.013*	–	–	4.43 (0.8–24.54)	0.09	0.41 (0.04–3.76)	0.43	4.13 (0.23–73.29)	0.33
Polyoma BK	24 (2.72–211.6)	0.004*	4.43 (0.8–24.54)	0.09	–	–	0.4 (0.08–1.85)	0.24	0.95 (0.06–16.28)	0.97
HCV	1.2 (0.25–5.77)	0.82	0.41 (0.04–3.76)	0.43	0.4 (0.08–1.85)	0.24	–	–	4.13 (0.23–73.29)	0.33
HBV	2.42 (0.14–41.87)	0.54	4.13 (0.23–73.29)	0.33	0.95 (0.06–16.28)	0.97	4.13 (0.23–73.29)	0.33	–	–
Laboratory										
Leukopenia	0.51 (0.13–2.02)	0.34	0.63 (0.14–2.96)	0.56	1.35 (0.4–4.57)	0.63	1.14 (0.26–5.03)	0.86	0 (0-.)	1.00
Lymphopenia	2.82 (0.71–11.2)	0.14	4.06 (0.88–18.86)	0.07	5 (1.14–22.02)	0.033*	1.2 (0.25–5.77)	0.82	2.42 (0.14–41.87)	0.54
Thrombocytopenia	0.83 (0.18–3.78)	0.80	0.79 (0.14–4.55)	0.80	1.2 (0.3–4.74)	0.80	3.09 (0.65–14.62)	0.16	NE	
Anemia	1.72 (0.43–6.85)	0.44	7.11 (0.8–63.24)	0.08	0.6 (0.17–2.07)	0.42	2.76 (0.5–15.29)	0.24	NE	
Creatinine (mg/dl)	0.84 (0.04–17.82)	0.91	0.22 (0–19.05)	0.50	0.31 (0.02–5.84)	0.43	19.88 (0.75–528.05)	0.07	2.67 (0.01–504.31)	0.71
Multivariate analysis for (viral infections/reactivations)										
Polyoma BK	24 (2.72–211.6)	0.004*	–		–		–	–		
Herpes simplex	–	–	–		24 (2.72–211.6)	0.004*	–	–		

*CI* confidence interval, *CNS* central nervous system, *HCV* hepatitis C virus, *HBV* hepatitis B virus, *NE* not estimated, *OR* odds ratio

*A *p* value of < 0.05 was considered to be statistically significant

## Discussion

Egypt, as a developing country, is highly prevalent in infectious diseases and the Egyptian low income population are more vulnerable to complications of infection especially immune-compromised patients. Many factors contribute to this like lack of sanitary infrastructure (e.g., water supply, sewage disposal system, and hospital hygiene). Food insecurity and malnutrition lead to weakened immune system. In addition, overcrowded households and hospitals facilitate transmission of pathogens [11].

In this study, the frequency of HSV viremia in pediatric ALL patients was (30.2%), parvo B19V (20.9%), polyoma BK (51.2%) and HBV (4.7%) and these percentages are high compared to a Japanese study that used Multiplex PCR in blood samples of immune-compromised patients and detected HSV1 in (0.9%**),** parvo B19V in (0.4%), BKV in (6.6%), and HBV in (0.7%) of all patients [12].

In the current study, the most frequent detected virus in blood of ALL patients was polyoma BK (51.2%) followed by herpes simplex (30.2%).

The same mentioned Japanese study found that cytomegalovirus (CMV) was the most frequent virus in allogeneic haematopoietic stem cell transplant (HSCT) patients, followed by HHV-6 [12]. A study in Tunisie also showed that CMV was the most frequently detected herpes family member in ALL patients followed by HHV-6 [13]. However, this is not the case in our institute as a previous study in 2018 reported absence of CMV infection either before diagnosis of malignancy, during hospitalization or even through induction phase of chemotherapy by virus reactivation [14].

In a previous study, BKV DNA was detected in (PBMCs) of (26.4%) of healthy blood donors [15]. Studies that examined the prevalence of BK virus in plasma of leukemia patients are limited in the literature. The frequency of polyoma BK DNA in a previous study was (28%) of blood samples of all cancer patients and (30%) of ALL cases compared to the higher frequency (51.2%) among ALL cases enrolled in our study [16].

HSV was highly prevalent in our study (30.2%). ALL patients were found to be highly exposed to herpes simplex infection both primary disease and reactivation [17, 18] but this varies according to the geographical area. A study in Tunisie did not detect any herpes simplex virus in acute leukemia cases and was detected in only (0.9%) of immunocompromised patients in Japan [12].

In the current study, parvovirus B19 DNA was detected in (20.9%) of patients. This is close to the results of previous studies [19, 20] but in both studies, detection of that virus was associated with cytopenia which is not the case in our study which is against its speculated role in the pathogenesis of ALL.

Frequency of HCV infection (20.9%) in this study was higher than that of HBV (4.7%) due to high endemicity in Egypt. A meta-analysis study in 2018 in Egypt has estimated pooled mean HCV prevalence to be 11.9% among the general population and 55.6% among populations at high risk (patients with repeated blood transfusion and/or patients on injections).[21] There is high rate of seroconversion of hepatitis B and C in pediatric malignancies in Egypt [9]. It was reported that 70% of ALL survivors were HCV positive and justified this by multiple blood and blood products transfusions during intensive therapy, frequent blood sampling, intravenous diagnostic procedures, intravenous fluid therapy and surgery [22]. Blood transfusion is still a major risk factor for hepatitis C infection despite strict screening of blood and blood products. It is supposed that screening for HCV by the standard tests (HCV antibodies) may be not sufficient, because anti-HCV antibodies are not present in some hepatitis C infected individuals. This is known as "sero-negative" HCV infection. Another condition called occult hepatitis C virus infection is common in leukemias. It is defined as the existence of hepatitis C RNA in the peripheral blood mononuclear cells or the liver without any detectable nucleic acid in serum. Occult hepatitis infection carries the risk of reactivation to clinically evident disease in cases of immune suppression. It should be noted that most hepatitis C patients in this study were relapsed cases which means that they received chemotherapy and blood transfusion in their first presentation. A recent study in Egypt has found that sero-negative RNA-positive hepatitis C patients represent 21% of all sero-negative leukemia cases and occult HCV represents 14% of them which imply a significant clinical problem in leukemic patients [23].

HBV shares all modes of transmission with HCV. Past infection with HBV is the major risk factor for HBV reactivation in Egypt and the incidence of HBV reactivation was 9.4% among hematological malignancies [24].

The prevalence of occult HBV infection was found to be higher in the leukemia patients than normal individuals [25]. Screening for occult hepatitis B&C by non-invasive methods is still challenging.

Further studies are needed to determine the infectivity of occult hepatitis B&C and determine the role of occult hepatitis B & C infected blood donors in transmission of hepatitis B & C especially to immunocompromised recipients, reconsider the efficiency of the standard screening methods and think about introducing molecular approaches in screening of blood donors in Egypt.

Co-infection of HBV and HCV was present in one case (0.023%). Co-infection with both viruses was reported previously in leukemia and lymphoma patients and was thought to play a role in the pathogenesis of hematologic malignancies as well as their role in hepatic cancer development [26].

In the current study, Viremia by multiple viruses was found in 34.9% of cases. Only one case was positive for five different viruses (1/43). The most frequent co-detected viruses were herpes simplex and polyoma BK (11.6%) followed by herpes simplex, parvo B19 and polyoma BK (9.3%).

Virus co-detection frequency is high in this study compared to a Japanese study in which multiple detection of two or more viruses was observed in 8.1% of immunocompromised patients, and co-infection with four kinds of viruses was observed in (3/2450) cases and the most frequent co-detection in all samples was a combination of CMV and Epstein–Barr virus (EBV) [12].

In this study, polyoma BK viremia was mainly associated with the occurrence of lymphopenia (*p* value 0.033). A previous study found that polyoma BK virus infection was associated with a decrease in the WBC in solid tumor patients [16]. Severe lymphopenia with absolute lymphocyte count (ALC) (< 500/μl) was found to be associated with co-infection with different herpes virus family members [13].

In this study, there was a significant association between polyoma BK viremia and herpes simplex viremia may be because both viruses can stay latent in human body and can be reactivated by the same state of immune suppression associated with ALL diagnosis taking into account that both viruses are highly prevalent in Egyptian population. Seroprevalence of herpes simples was 97.5% among Egyptians [27]. The prevalence of polyoma BK in stool samples of Egyptians was 31.7% (19/60) [28].

In the literature, the central nervous system (CNS) is the most frequently affected extramedullary site at diagnosis (< 5%) and at relapse (up to 30–40%) and its occurrence indicates bad prognosis [29]**. **In this study, CNS leukemia was present in (34.9%) of both new and relapsed leukemia patients, but incidence of CNS leukemia was significantly associated with HCV infection (*p* value 0.034). HCV infection was associated with a five-fold increase of odds of having CNS leukemia than those without hepatitis C infection. To the best of our knowledge, our study is the first to find a link between hepatitis C infection and CNS leukemia. It can be explained theoretically by the ability of HCV to stimulate B cell clonal expansion and cause high WBC count which is a risk factor of CNS leukemia [30]. A previous study reported that HCV patients are at higher risk of developing lymphoproliferative disorders for example: acute lymphoid leukemia B cell (early pre B type) and B&T cell lymphoma [26]. We formulated another theory that may explain the association between Hepatitis C and CNS leukemia in which adhesion molecules are the suspect. It is known that CNS invasion is related to adhesion molecules expressed by a subpopulation of leukemic cells that make them sticky and able to interact and adhere to the endothelial cells of the blood brain barrier. Examples of these adhesion molecule are (CD56/NCAM, VLA-4, ICAM-1, VCAM, L-selectin, LFA-, CD44, CXCL12). ICAM-1 interactions with the β2 integrins located on the surface of leukocytes are important for their firm adhesion to the endothelium.Soluble ICAM-1 can bind to LFA-1 and block its sites on effector cells inhibiting antitumor response and promote tumor progression [31]. Soluble adhesion molecules, because of their proteolytic activity and cell signaling functions was considered as tumor progressive factors [32]. Some of them was used to monitor leukemia relapse [33].

Chronic hepatitis C patients showed significant increase in soluble adhesion molecules, sICAM-1, sVCAM-1 as well as TNF-α [34–36]. Cytokines like TNF- induce an increase in ICAM-1 and VCAM-1 expression on endothelial cells [37].

VEGF showed dramatically elevated serum levels in HCV-positive cases [38]. The VEGF levels in CSF but not plasma were significantly increased in patients with ALL with CNS involvement [39].

Further prospective studies on soluble and cell bound ICAM-1 and VCAM-1and other adhesion molecules in ALL patients with hepatitis C infection are needed to confirm the possibility that hepatitis C infection is a new risk factor of incidence of CNS leukemia and search for direct causes that made this association.

Viral infections in our study were not associated with bad clinical conditions (Table 5) so, it is not recommended to hold chemotherapy in viral infections except in severe life threatening cases. Instead, specific and supportive treatment should be started with.

In herpes simplex infection suspected clinically, acyclovir is added to treatment regimen. Patients with severe anemia associated with parvovirus B19 infection should receive blood transfusion or intravenous immunoglobulin. As a consequence of this, screening of all patients for these viruses is not recommended. But in hepatitis virus infections, chemotherapy regimen should be modified according to liver condition. The new DAAS drugs (sofosbuvir and velpatasvir) for hepatitis C should be administrated to patients older than 12 or more than 35 kg in weight according to FDA guidelines [40]. Treatment of younger children and timing of administration of DAAS during or after chemotherapy are still under research.

This study had limitations such as the small number of patients enrolled, larger epidemiological studies are needed to confirm the mentioned associations. HSV and polyoma BK viremia may represent primary infection or recurrent infection by reactivation. Further studies involving ELISA testing of IgM and IgG antibodies are needed to differentiate between both types of infection.

In conclusion, there is a high frequency of viremia by single virus and viremia by multiple viruses at the time of diagnosis of acute lymphoblastic leukemia in pediatrics. Polyoma BK is the most common detected virus and is mainly associated with lymphopenia. It was also significantly associated with herpes simplex viremia. HCV infection was associated with increased incidence of CNS leukemia.

## Supplementary Information

Below is the link to the electronic supplementary material.

Supplementary file1 (XLSX 17 KB)

Supplementary file2 (DOCX 23 KB)

## Data Availability

The original contributions presented in the study are included in the supplementary material. nESM1: Research data file.ESM2: characteristics of ALL cases in different viral infections. Further inquiries can be directed to the corresponding authors.

## Abbreviations

ALL: Acute lymphoblastic leukemia patients
ALT: Alanine aminotransferase
AST: Aspartate aminotransferase
CBC: Complete blood picture
CMV: Cytomegalovirus
CNS: Central nervous system
DAAS: Direct acting antiviral drugs
EBV: Epstein–Barr virus
HBV: Hepatitis B virus
HCV: Hepatitis C virus
HSV: Herpes simplex virus
PBMCs: Peripheral blood mononuclear cells
qRT-PCR: Quantitative reverse transcription polymerase chain reaction
SARS-COV2: Severe acute respiratory syndrome corona virus 2
SECI: South Egypt Cancer Institute
SGPT: Serum glutamic pyruvic transaminase
SGOT: Serum glutamic oxaloacetic transaminase
