# Supplementary material for: Investigating causes and risk factors of pre-chemotherapy viremia in acute lymphoblastic leukemia pediatric patients
Source: Infection. 2022 Jul 25;51(1):203–11. doi: 10.1007/s15010-022-01878-9 (PMC9309998; doi:10.1007/s15010-022-01878-9)
Supplement: Supplementary file 2 — Supplementary file2 (DOCX 23 KB) [file 15010_2022_1878_MOESM2_ESM.docx]

**Table 6 : Demographical, clinical and laboratory characteristics of ALL cases in different viral infections.**

|  | | **Herpes simplex** | | | | **ParvoB19** | | | | **Polyoma BK** | | | | **HCV** | | | | **HBV** | | | |
| --- | --- | --- | --- | --- | --- | --- | --- | --- | --- | --- | --- | --- | --- | --- | --- | --- | --- | --- | --- | --- | --- |
|  | | **Positive** | | **Negative** | | **Positive** | | **Negative** | | **Positive** | | **Negative** | | **Positive** | | **Negative** | | **Positive** | | **Negative** | |
|  | | (N=13) | | (N=30) | | (N=9) | | (N=34) | | (N=22) | | (N=21) | | (N=9) | | (N=34) | | (N=2) | | (N=41) | |
| Age (y) | | 7 (3-15) | | 7 (2-14) | | 9 (5.5-15) | | 7 (2-14) | | 7 (2-15) | | 7 (2-14) | | 7 (5-15) | | 7 (2-15) | | 13 (11-15) | | 7 (2-15) | |
| Sex | |  | |  | |  | |  | |  | |  | |  | |  | |  | |  | |
| Male | | 8(61.5%) | | 18(60.0%) | | 7(77.8%) | | 19(55.9%) | | 11(50.0%) | | 15(71.4%) | | 6(66.7%) | | 20(58.8%) | | 2(100.0%) | | 24(58.5%) | |
| Female | | 5(38.5%) | | 12(40.0%) | | 2(22.2%) | | 15(44.1%) | | 11(50.0%) | | 6(28.6%) | | 3(33.3%) | | 14(41.2%) | | 0(0.0%) | | 17(41.5%) | |
| Subtype | |  | |  | |  | |  | |  | |  | |  | |  | |  | |  | |
| B-cell | | 9(69.2%) | | 24(80.0%) | | 6(66.7%) | | 27(79.4%) | | 15(68.2%) | | 18(85.7%) | | 6(66.7%) | | 27(79.4%) | | 1(50.0%) | | 32(78.0%) | |
| T-cell | | 4(30.8%) | | 6(20.0%) | | 3(33.3%) | | 7(20.6%) | | 7(31.8%) | | 3(14.3%) | | 3(33.3%) | | 7(20.6%) | | 1(50.0%) | | 9(22.0%) | |
| Status of disease | |  | |  | |  | |  | |  | |  | |  | |  | |  | |  | |
| Denovo | | 3(23.1%) | | 7(23.3%) | | 3(33.3%) | | 7(20.6%) | | 5(22.7%) | | 5(23.8%) | | 1(11.1%) | | 9(26.5%) | | 0(0.0%) | | 10(24.4%) | |
| Relapse | | 10(76.9%) | | 23(76.7%) | | 6(66.7%) | | 27(79.4%) | | 17(77.3%) | | 16(76.2%) | | 8(88.9%) | | 25(73.5%) | | 2(100.0%) | | 31(75.6%) | |
| CNS involvement  With leukemia | | 6(46.2%) | | 9(30.0%) | | 3(33.3%) | | 12(35.3%) | | 5(22.7%) | | 10(47.6%) | | 6(66.7%) | | 9(26.5%) | | 1(50.0%) | | 14(34.1%) | |
| GIT Mucositis | | 8(61.5%) | | 3(10.0%) | | 4(44.4%) | | 7(20.6%) | | 8(36.4%) | | 3(14.3%) | | 3(33.3%) | | 8(23.5%) | | 1(50.0%) | | 10(24.4%) | |
| Urinary symptoms | | 3(23.1%) | | 1(3.3%) | | 2(22.2%) | | 2(5.9%) | | 4(18.2%) | | 0(0.0%) | | 1(11.1%) | | 3(8.8%) | | 1(50.0%) | | 3(7.3%) | |
| Herpes simplex | | - | | - | | 6(66.7%) | | 7(20.6%) | | 12(54.5%) | | 1(4.8%) | | 3(33.3%) | | 10(29.4%) | | 1(50.0%) | | 12(29.3%) | |
| ParvoB19 | | 6(46.2%) | | 3(10.0%) | | - | | - | | 7(31.8%) | | 2(9.5%) | | 1(11.1%) | | 8(23.5%) | | 1(50.0%) | | 8(19.5%) | |
| Polyoma BK | | 12(92.3%) | | 10(33.3%) | | 7(77.8%) | | 15(44.1%) | | - | | - | | 3(33.3%) | | 19(55.9%) | | 1(50.0%) | | 21(51.2%) | |
| HCV | | 3(23.1%) | | 6(20.0%) | | 1(11.1%) | | 8(23.5%) | | 3(13.6%) | | 6(28.6%) | | - | | - | | 1(50.0%) | | 8(19.5%) | |
| HBV | | 1(7.7%) | | 1(3.3%) | | 1(11.1%) | | 1(2.9%) | | 1(4.5%) | | 1(4.8%) | | 1(11.1%) | | 1(2.9%) | | - | | - | |
| COVID-19 | | 1(7.7%) | | 2(6.7%) | | 2(22.2%) | | 1(2.9%) | | 3(13.6%) | | 0(0.0%) | | 0(0.0%) | | 3(8.8%) | | 0(0.0%) | | 3(7.3%) | |
| WBCs (x10^3/mL) count | 6.3 (3-384) | | 6.2 (3-390) | | 23 (3-333) | | 6.2 (3.2-390) | | 5 (3-390) | | 8 (3-250) | | 5 (3.4-270) | | 6.5 (3-390) | | 5.7 (5-6.4) | | 6.3 (3-390) | |  |
| Leukopenia | | 4(30.8%) | | 14(46.7%) | | 3(33.3%) | | 15(44.1%) | | 10(45.5%) | | 8(38.1%) | | 4(44.4%) | | 14(41.2%) | | 0(0.0%) | | 18(43.9%) | |
| Lymphocytes count | | 2 (0.3-200) | | 3 (0.5-286) | | 1.3 (0.3-286) | | 3 (0.5-230) | | 2 (0.3-286) | | 3.6 (0.7-150) | | 3 (0.3-104) | | 3 (0.5-286) | | 2 (0.3-3.6) | | 3 (0.5-286) | |
| Lymphopenia | | 6(46.2%) | | 7(23.3%) | | 5(55.6%) | | 8(23.5%) | | 10(45.5%) | | 3(14.3%) | | 3(33.3%) | | 10(29.4%) | | 1(50.0%) | | 12(29.3%) | |
| Platelets (x10^3/mL) | | 189 (66-416) | | 253.5 (24-905) | | 200 (133-345) | | 253.5 (24-905) | | 195 (24-905) | | 255 (36-488) | | 219 (36-488) | | 271.5 (24-905) | | 252 (249-255) | | 219 (24-905) | |
| Thrombocytopenia | | 3(23.1%) | | 8(26.7%) | | 2(22.2%) | | 9(26.5%) | | (27.3%) | | 5(23.8%) | | 4(44.4%) | | 7(20.6%) | | 0(0.0%) | | 11(26.8%) | |
| Hb (g/dl) | | 11 (5.5-14) | | 10.6 (7.5-14) | | 11.5 (5.5-14) | | 10.4 (7.5-14) | | 10.8 (5.5-14) | | 11.5 (8-14) | | 11.2 (9-14) | | 11 (5.5-14) | | 12.9 (11.7-14) | | 11 (5.5-14) | |
| Anemia<10g/dl | | 4(30.8%) | | 13(43.3%) | | 1(11.1%) | | 16(47.1%) | | (45.5%) | | 7(33.3%) | | 2(22.2%) | | 15(44.1%) | | 0(0.0%) | | 17(41.5%) | |
| SGOT (AST) | | 43 (29-243) | | 36 (15-170) | | 33 (23-168) | | 38 (15-243) | | 38 (18-243) | | 36 (15-170) | | 105 (35-243) | | 36 (15-168) | | 53.5 (40-67) | | 36 (15-243) | |
| High SGOT | | 10(76.9%) | | (66.7%) | | 5(55.6%) | | 25(73.5%) | | (68.2%) | | 15(71.4%) | | 9(100.0%) | | 21(61.8%) | | 2(100.0%) | | 28(68.3%) | |
| SGPT (ALT) | | 41 (19-230) | | 33 (7-226) | | 33 (24-175) | | 34.5 (7-230) | | 33.5 (12-230) | | 34 (7-226) | | 136 (38-230) | | 31 (7-175) | | 67 (14-120) | | 34 (7-230) | |
| High SGPT | | 5(38.5%) | | (33.3%) | | 2(22.2%) | | 13(38.2%) | | (31.8%) | | 8(38.1%) | | 8(88.9%) | | 7(20.6%) | | 1(50.0%) | | 14(34.1%) | |
| Creatinine (mg/dl) | | 0.3 (0.2-0.7) | | 0.3 (0.1-1.2) | | 0.3 (0.2-0.5) | | 0.3 (0.1-1.2) | | 0.3 (0.2-0.7) | | 0.3 (0.1-1.2) | | 0.4 (0.2-1.1) | | 0.3 (0.1-1.2) | | 0.4 (0.4-0.4) | | 0.3 (0.1-1.2) | |
| High creatinine | | 0(0.0%) | | (6.7%) | | 0(0.0%) | | 2(5.9%) | | (0.0%) | | 2(9.5%) | | 1(11.1%) | | 1(2.9%) | | 0(0.0%) | | 2(4.9%) | |

AST: Aspartate aminotransferase, ALT: Alanine aminotransferase
